# Benchmarking quantum versions of the kNN algorithm with a metric based on amplitude-encoded features

**DOI:** 10.1038/s41598-024-67392-0

**Published:** 2024-07-19

**Authors:** Areli-Yesareth Guerrero-Estrada, L. F. Quezada, Guo-Hua Sun

**Affiliations:** 1 Computing Research Center, National Polytechnic Institute, 07700 Mexico City, Mexico; 2 Research Center for Quantum Physics, Huzhou University, Huzhou, 313000 People’s Republic of China

**Keywords:** Amplitud-encoded features, Quantum machine learning, Quantum kNN, Quantum metric, Computational science, Information technology, Quantum information, Qubits

## Abstract

This work introduces a quantum subroutine for computing the distance between two patterns and integrates it into two quantum versions of the kNN classifier algorithm: one proposed by Schuld et al. and the other proposed by Quezada et al. Notably, our proposed subroutine is tailored to be memory-efficient, requiring fewer qubits for data encoding, while maintaining the overall complexity for both QkNN versions. This research focuses on comparing the performance of the two quantum kNN algorithms using the original Hamming distance with qubit-encoded features and our proposed subroutine, which computes the distance using amplitude-encoded features. Results obtained from analyzing thirteen different datasets (Iris, Seeds, Raisin, Mine, Cryotherapy, Data Bank Authentication, Caesarian, Wine, Haberman, Transfusion, Immunotherapy, Balance Scale, and Glass) show that both algorithms benefit from the proposed subroutine, achieving at least a 50% reduction in the number of required qubits, while maintaining a similar overall performance. For Shuld’s algorithm, the performance improved in Cryotherapy (68.89% accuracy compared to 64.44%) and Balance Scale (85.33% F1 score compared to 78.89%), was worse in Iris (86.0% accuracy compared to 95.33%) and Raisin (77.67% accuracy compared to 81.56%), and remained similar in the remaining nine datasets. While for Quezada’s algorithm, the performance improved in Caesarian (68.89% F1 score compared to 58.22%), Haberman (69.94% F1 score compared to 62.31%) and Immunotherapy (76.88% F1 score compared to 69.67%), was worse in Iris (82.67% accuracy compared to 95.33%), Balance Scale (77.97% F1 score compared to 69.21%) and Glass (40.04% F1 score compared to 28.79%), and remained similar in the remaining seven datasets.

## Introduction

Machine learning, as a subfield of artificial intelligence, focuses on developing algorithms and models capable of learning from data without explicit programming. This characteristic enables them to adapt and improve performance automatically as they gain exposure to more information. Consequently, they can identify patterns, extract meaningful insights, and make predictions or decisions.

In supervised learning, algorithms are trained using labeled datasets, where each pattern is associated with a known class label. This methodology allows the algorithm to learn the relationship between input features and corresponding labels. Supervised learning algorithms encompass multiple methods. For instance, in 1952, Fix and Hodges introduced the k-nearest neighbors (kNN) algorithm ^1^, which utilizes the distance between patterns to assign class labels. In the same decade, Rosenblatt ^2^ presented the perceptron, a fundamental neural network model that adjusts weights for classification tasks. Another significant development occurred in 1986 when Quinlan proposed the Iterative Dichotomiser 3 (ID3) algorithm, a model based on decision trees ^3^, which divides data into branches based on relevant features. Subsequently, in 1995, Vapnik and Chervonenkis presented support vector machines (SVM) ^4^, aiming to find optimal hyperplanes in a space to separate data from different classes. These algorithms found numerous applications across various fields, including computer vision, image, and speech recognition ^5–7^, natural language processing ^8–10^, recommendation systems ^11,12^, fraud detection ^13–15^, healthcare ^16,17^, finance ^18–20^, among others.

On the other hand, in recent years, quantum computing has emerged as a new attractive field that leverages quantum phenomena such as entanglement and superposition to efficiently solve complex mathematical problems that traditional computers may struggle with or find unfeasible to solve. This new paradigm has opened opportunities for developing algorithms that demonstrate superiority over classical computation in specific tasks, such as factorizing large numbers, simulating quantum systems, and optimizing complex systems ^21–24^. Naturally, quantum computing began to be applied to machine learning tasks, giving rise to the field now known as Quantum Machine Learning (QML). Thanks to quantum parallelism, tasks that were challenging for classical computers can be executed more efficiently on quantum computers, instilling optimism about the potential of QML ^25^.

To date, several quantum and quantum-classical hybrid versions of classical machine learning algorithms have been proposed, including quantum neural networks (QNN) ^26–29^, quantum associative memories (QAM) ^30^, quantum support vector machine (QSVM) ^31,32^, and quantum k-nearest neighbors algorithm (QkNN) ^25,33–35^. Quantum approaches have demonstrated effectiveness in various applications such as image classification ^26,36,37^, text processing ^38^, medical applications ^39–43^, data mining ^44^ and financial applications ^45^. QkNN algorithms, in particular, use quantum circuits for distance calculations. This method can reduce time complexity from polynomial to logarithmic scales in some scenarios, making feasible to analyze certain datasets where the classical kNN is computationally too demanding ^44,46,47^. However, as we will show, there is still room for improvement, particularly in reducing the number of qubits required by these algorithms to encode numerical features. This reduction can potentially make them easier to implement on NISQ computers.

In this paper, we explore the modification of two such QkNN models ^25,33^ to further optimize their efficiency and memory requirements. Instead of the originally used Hamming metric ^48^ with qubit-encoded features, this work proposes a quantum subroutine that computes the distance between patterns using amplitude-encoded features. To assess the performance of the modified algorithms, we conduct a thorough analysis using thirteen numerical datasets. Our objective is to evaluate the effectiveness and impact of these modified algorithms in classification tasks, offering a promising alternative to metric-based quantum algorithms.

This paper is organized as follows: Section 2 provides some basic definitions and descriptions of the subroutines and the quantum kNN versions proposed by Schuld and Quezada. Section 3 gives the details of the proposed metric and the corresponding adaptations of the quantum kNN algorithms incorporating the proposed subroutine. The results of the computational experiments and the comparison between the two algorithms are presented in Section 4. Lastly, Section 5 concludes the paper and discusses potential future work.

## Background

### Classical kNN

One common approach in supervised learning involves using distances to classify patterns. These distances, such as the Euclidean or the Hamming metrics, quantify the similarity or dissimilarity between patterns. The underlying assumption is the elements of the same class are more likely to exhibit similarities and be closer to each other in the features space. By calculating the distances between patterns and using predefined decision boundaries or thresholds, an algorithm can assign a class to an unlabeled pattern.

One of the most popular metric-based algorithms is the kNN ^1^. Due to its simplicity, it is widely used on different datasets, including images and texts ^49^. Furthermore, its implementation can employ different classical, quantum, and hybrid (quantum-classical) approaches ^50,51^.

Consider a training set of binary n-dimensional patterns $T=\{ (x^1,c^1),\dots ,(x^N,c^N) \}$ where $x^j=[ 0,1 ]_n$ and an unlabeled pattern $x^k$ will be classified. The classical version of the kNN algorithm consists of the following steps:

- The algorithm stores the training patterns and their corresponding class labels in the training phase.
- Subsequently, in the classification phase, the algorithm calculates the distance between a given pattern to be classified, denoted as $x^k$, and each pattern element of the training set *T*.
- Lastly, the algorithm assigns the majority class among the *k* closest elements based on the computed distances. In the case of ties, a rule such as selecting the class with the smallest mean distance must be predefined.

The choice of the distance metric and the value of *k* can significantly affect the performance of the kNN algorithm. Different distance metrics may be more suitable for different data types and domains. Even though the kNN algorithm is simple and easy to implement, it can be computationally expensive, specially for large datasets, as it requires calculating the distance to all training samples for every prediction. The resulting complexity is thus O(*nN*), where *n* is the number of features (the dimension of the pattern), and *N* is the number of patterns in the training set.

### Schuld’s quantum version

The quantum kNN algorithm proposed by Schuld et al. ^25^ initializes the patterns in the training set into an equiprobable superposition

1 $$\begin{aligned} \mid \psi _0 \rangle = \frac{1}{\sqrt{N}} \sum \limits _{j=1}^{N} \mid x^k;x^j;c^j;0 \rangle . \end{aligned}$$

The similarity between features is stored in the qubits associated with the training patterns $\mid x^j \rangle$, and a special unitary operator $U_f$ is used to encode the corresponding Hamming distance $d_{h}$ in the amplitude of each element in the superposition, resulting in an output state of the form:

2 $$\begin{aligned} \mid \psi _f \rangle = \frac{1}{\sqrt{N}} \sum \limits _{j=1}^{N} \cos \left( \beta ^j \right) \mid x^k;d^{j};c^j;0 \rangle +\frac{1}{\sqrt{N}} \sum \limits _{j=1}^{N} \sin \left( \beta ^j \right) \mid x^k;d^{j};c^j;1 \rangle , \end{aligned}$$

where $$\beta ^j = \displaystyle \frac{\pi \cdot d_h(x^k;x^j)}{2n}$$ and

3 $$\begin{aligned} d^{j}_{i} = \left\{ \begin{matrix} 1 &{} \text {if} \quad x^{k}_{i} = x^{j}_{i} ,\\ \\ 0 &{} \text {if} \quad x^{k}_{i} \ne x^{j}_{i} . \end{matrix} \right. \end{aligned}$$

**Figure 1:**
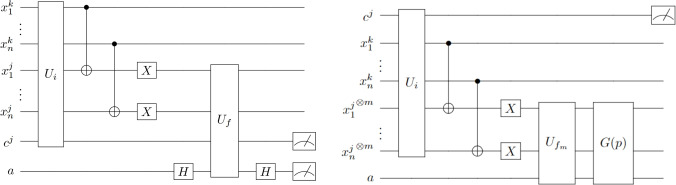
Left: Quantum circuit of Schuld’s algorithm. $U_i$ represents the initialization phase, and $U_f$ denotes the unitary operator that encodes the Hamming distance between patterns in the amplitude of the corresponding element in the superposition. Right: Quantum circuit of Quezada’s algorithm. $U_i$ represents the initialization phase, $U_{f_m}$ denotes the sorting algorithm and *G*(*p*) the *p* applications of the Grover subroutine.

Figure 1 shows the circuit associated with Schuld’s algorithm.

Notice that the term where the last qubit is $\mid 0 \rangle$ in Eq. (2) is the one where it is more likely to measure a class corresponding to one of the nearest neighbors. This is due to the amplitude being $\cos (\beta _{j})$, and $\beta _{j}$ being proportional to the Hamming distance between patterns. That is, if the neighbors are near, then $\beta _{j} \approx 0$ and thus $\cos (\beta _{j}) \approx 1$. On the other hand, the term where the last qubit is $\mid 1 \rangle$, the sine amplitude amplifies the opposite probability, that is the one where the class corresponds to one of the farthest neighbors.

Schuld et al. propose to run the algorithm *t* times, where *t* is a previously defined threshold such that $t > k$. For each execution, the ancilla qubit is measured. If $\mid 0 \rangle$ is obtained, the class is also measured; if $\mid 1 \rangle$ is measured, the execution is discarded. This process is repeated until the *k* neighbors or threshold *t* are reached. The class that appears the most among the *k* (or less) candidates is selected. Analogous to the classical version, tie-breaking rules must be defined beforehand.

The probability of measuring the ancilla qubit at $\mid 0 \rangle$ is given by

4 $$\begin{aligned} P_0 = \frac{1}{N} \sum \limits _{j=1}^{N} \cos ^2\left( \beta ^{j} \right) . \end{aligned}$$

So, the probability of obtaining a specified class *c* is given by

5 $$\begin{aligned} P(c) = \frac{1}{P_0 N} \sum \limits _{j\mid x^j\in c}^{N} \cos ^2\left( \beta ^{j} \right) . \end{aligned}$$

It is worth mentioning that this algorithm is strongly based on the Hamming distance, thus requiring classical data to be binarized and encoded in qubits. If the analyzed dataset has *f* features, each feature requires an average of $\bar{\alpha }$ qubits to encode the corresponding numerical values and *c* represents the number of qubits required to encode the class, then the algorithm necessitates at least

6 $$\begin{aligned} N^{\text {S}}_{\text {original}} = 2\bar{\alpha }f+c+1 \end{aligned}$$

qubits in order to be implemented (ignoring initialization).

### Quezada’s quantum version

The quantum kNN algorithm proposed by Quezada et al. ^33^ is based on the (*m*, *p*) sorting algorithm, where *m* is the length of the array to be sorted and $p \in \mathbb {N}$ corresponds to the times that the Grover subroutine is applied. The initial state is prepared as follows:

7 $$\begin{aligned} \mid \psi _0 \rangle = \frac{1}{\sqrt{N}} \sum _{j=1}^{N} \mid c^{j};x^{k};x^{j} \rangle \otimes \mid T_x \rangle ^{\otimes (m-1)} \otimes \mid 0 \rangle , \end{aligned}$$

where $$\mid T_x \rangle = \displaystyle \frac{1}{\sqrt{N}} \sum _{j=1}^{N} \mid x^{j} \rangle .$$

As in Schuld’s version, the second step consists of computing the features’ similarities between the training patterns $x^{j}$ and the unlabeled pattern $x^{k}$ and storing them in the qubits associated with the training patterns. The (*m*, *p*) sorting algorithm is then applied to these qubits, which at this point of the algorithm are in the state $\mid d^j\rangle ^{\otimes m}\otimes \mid 0 \rangle$, where $d^{j}_{i}$ is as in Eq. (3).

The resulting final state is given by

8 $$\begin{aligned} \mid \psi _f \rangle = \frac{\cos [(2p+1)\theta ]}{\sqrt{\nu }} \sum \limits _{ \begin{matrix} {j_1},...,{j_m} \\ \text {No ord} \end{matrix} }^{N} \mid c^{j_{1}};x^k;d^{j_1}...d^{j_m};0 \rangle + \frac{\sin [(2p+1)\theta ]}{\sqrt{\mu }} \sum \limits _{ \begin{matrix} {j_1},...,{j_m} \\ \text {Ord} \end{matrix} }^{N} \mid c^{j_{1}};x^k;d^{j_1}...d^{j_m};1 \rangle , \end{aligned}$$

where

9 $$\begin{aligned} \mu&= \displaystyle \frac{N!}{m!(N-m)!}, \end{aligned}$$

10 $$\begin{aligned} \nu&= N^m - \mu ,\end{aligned}$$

11 $$\begin{aligned} \theta&= \arcsin \left( \displaystyle \sqrt{\frac{\mu }{N^m}}\right) , \end{aligned}$$

and “No ord”, “Ord” stand for “non ordered” and “ordered” respectively. This order is the one performed by the (*m*, *p*) sorting algorithm, which tags those registers that do not respect $d^{j_1}< \cdots < d^{j_m}$, where the relation < is based on the number of 1’s in each $d^{j_i}$. Figure 1 shows the circuit associated with Quezada’s algorithm.

The last step is simply measuring the class qubit $\mid c^j \, \rangle$, and adding it as one of the *k* possible candidates. The probability of getting an arbitrary class *c* is given by

12 $$\begin{aligned} P(c) = \frac{\cos ^{2}[(2p+1)\theta ]}{\nu } \sum \limits _{x \in c} N_0(x) + \frac{\sin ^{2}[(2p+1)\theta ]}{\mu } \sum \limits _{x \in c} N_1(x), \end{aligned}$$

where,

13 $$\begin{aligned} N_0(x)= & {} \left\{ \begin{matrix} N^{m-1} &{} if\, x < m, \\ N^{m-1} - \frac{(x-1)!}{(m-1)!(x-m)!} &{} if\, x \ge m, \end{matrix} \right. \end{aligned}$$

14 $$\begin{aligned} N_1(x)= & {} \left\{ \begin{matrix} 0 &{} if\, x < m, \\ \frac{(x-1)!}{(m-1)!(x-m)!} &{} if\, x \ge m. \end{matrix} \right. \end{aligned}$$

Lastly, the whole process is repeated *k* times to obtain the *k* class candidates.

Under the assumptions that $N \gg m$ and $\arcsin {\frac{1}{\sqrt{m!}}} \approx \frac{1}{\sqrt{m!}}$, the relationship between *m* and *p* that optimizes the algorithm is given by,

15 $$\begin{aligned} (2p+1)\sqrt{\frac{1}{m!}} \approx \frac{\pi }{2} (2 w + 1), \end{aligned}$$

where *w* is an integer. It should also be considered that $p \in \mathbb {N}$ represents the number of times that the Grover subroutine is applied. The optimal value of *p* as a function of *m* is thus

16 $$\begin{aligned} p_{\text {opt}} \approx \frac{\pi }{4}\sqrt{m!}-\frac{1}{2}. \end{aligned}$$

As in Schuld’s version, Quezada’s also uses the Hamming distance to compare patterns, and thus, its implementation requires numerical data to be binarized. In this case, if the analyzed dataset has *f* features, each feature requires an average of $\bar{\alpha }$ qubits to encode the corresponding numerical values and *c* represents the number of qubits required to encode the class, then the algorithm necessitates at least

17 $$\begin{aligned} N^{\text {Q}}_{\text {original}} = (m+1)\bar{\alpha }f+c+1 \end{aligned}$$

qubits in order to be implemented (ignoring initialization).

## QkNN algorithm with a non-binary similarity between features

### Metric based on amplitude-encoded features

This section introduces a subroutine that calculates the similarity between features of distinct patterns, and subsequently, this information will be used to compute the distance between them.

When conducting real data analysis, it is necessary to perform preprocessing steps to prepare the data. For numerical data, normalization between the range of 0 and 1 is required, while categorical data needs to be encoded as binary numbers. In this context, let us consider a training set consisting of n-dimensional numerical patterns denoted as $T=\left\{ \left( x^1,c^1\right) ,\dots ,\left( x^N,c^N\right) \right\}$, where $x^{j} = \left( x^{j}_{1},\dots , x^{j}_{n} \right)$. Additionally, we have an unlabeled pattern $x^k$ that needs to be classified.

Computing the Hamming distance, which is employed in both Schuld’s and Quezada’s QkNN algorithms, requires the utilization of CNOT gates to compare the qubit-encoded features of the test pattern $x^{k}$ with those of the training patterns $x^{j}$. The comparison information, termed as $d^{j}$ in Eq. (3), is then stored in the qubits corresponding to the training patterns, effectively deleting the original data in the process. This can be clearly seen in Eqs. (2) and (8), where the qubits corresponding to the training patterns $x^{j}$ have been replaced by $d^{j}$.

Here, we present an alternative similarity measure that eliminates the need for binarizing numerical data. Furthermore, our proposed method reduces the number of qubits needed to implement the algorithms, as it only requires a single qubit per numerical feature. The computation of the proposed similarity between features involves applying a rotation around the y-axis on a single qubit initialized in $\mid 0 \rangle$. This rotation employs the difference between the numerical values of the corresponding features as the angle of rotation. The resulting quantum state compares each feature of $x^k$ with all the patterns from the training set:

18 $$\begin{aligned} \mid d^{j} \rangle = \bigotimes \limits _{i=1}^{n} \left[ \cos \left( \frac{\pi \lambda _i^j}{2} \right) \mid 0 \rangle + \sin \left( \frac{\pi \lambda _i^j}{2} \right) \mid 1 \rangle \right] \end{aligned}$$

with $\lambda _i^j = x_i^j - x_i^k$ and $j \in \{1,2,...,n\}$. Notice that Eq. (18) can also be written in the more convenient form

19 $$\begin{aligned} \mid d^j \rangle = \sum \limits _{g=0}^{2^n-1} \gamma _g^j \mid g \rangle , \end{aligned}$$

where

20 $$\begin{aligned} \gamma _g^j = \prod \limits _{i=1}^{n} \sin \left[ \frac{\pi }{2}\left( x_{i}^{j}-x_{i}^{k} + g_{i}\right) \right] , \end{aligned}$$

and $g_{i}$ denotes the *i*-th binary digit of *g*.

The transition from $\mid 0 \, \rangle ^{\otimes n}$ to $\mid d^{j} \, \rangle$ can be unitarily done using a set of controlled rotations. These rotations need to have the corresponding angles encoded in them, and the controlling qubits must uniquely identify each pattern in the training set, similar to having an index register. Expressing the index register of $\mid x^{j} \rangle$ as $\mid j \rangle$, the Eq. (21) describes the unitary operator, which we will denote as $U_{i}$, satisfies

21 $$\begin{aligned} U_{i} \mid j \rangle \otimes \mid 0 \rangle ^{\otimes n} = \mid j \rangle \otimes \bigotimes _{w=1}^{n} \left[ R_{y} \left( \frac{\pi }{2}\cdot x_{w}^{j} \right) \mid 0 \rangle \right] . \end{aligned}$$

Afterwards, the comparison with the features of $x^{k}$ is performed by rotating the same *n* qubits in the opposite direction using the operator $R_{y} \left[ -\frac{\pi }{2}\left( x_{w}^{k}\right) \right]$, which results in the state $\mid d^{j} \, \rangle$ from Eq. (19).

Notice that the cosine term in equation Eq. (18) quantifies the similarity between the features. $\left( x_{w}^{j}-x_{w}^{k} \right) \rightarrow 0$ indicates a high degree of similarity, resulting in a cosine value close to one. Conversely, if the corresponding features are significantly different, the sine term becomes dominant. Furthermore, if the patterns are binary, then $\cos \left( \frac{\pi \lambda _{k}}{2}\right)$ is equivalent to the binary similarity outlined in Eq. (3).

### Schuld’s QkNN modified algorithm

The algorithm implements the similarity measure discussed in the former subsection and modifies the QkNN algorithm proposed by Schuld et al. in the following way:

- The initial step of the algorithm is preparing the pattern superposition
  22 $$\begin{aligned} \mid \psi _0 \rangle = \frac{1}{\sqrt{N}} \sum \limits _{j=1}^{N} \mid j; c^j \rangle \otimes \mid 0 \rangle ^{\otimes n} \otimes \mid 0 \rangle , \end{aligned}$$
  where *N* is the number of patterns in the training set, *n* is the number of features in each pattern, and $\mid j \, \rangle$ is an index register for the pattern $x^j$.
- The next step involves applying the $U_{i}$ operator described in Eq. (21), which rotates the *n* qubits $\mid 0 \rangle ^{\otimes n}$ based on the *n* features associated with each pattern in the training dataset. To differentiate the features belonging to each specific pattern, these rotations must be controlled through the index register qubits $\mid j \rangle$, which provide unique identification for each pattern in the training dataset. Subsequently, the comparison with the features of $x^{k}$ is performed by rotating the same *n* qubits in the opposite direction using the operator $R_{y} \left[ -\frac{\pi }{2}\left( x_{w}^{k}\right) \right]$. The resulting state is given by
  23 $$\begin{aligned} \mid \psi _1 \rangle = \frac{1}{\sqrt{N}} \sum \limits _{j=1}^{N} \left[ \mid j; c^j \rangle \otimes \mid d^j \rangle \right] \otimes \mid 0 \rangle , \end{aligned}$$
  where $\mid d^j \rangle$ is described as in Eq. (19).
- Apply a Hadamard gate to the ancilla qubit, resulting in
  24 $$\begin{aligned} \mid \psi _2 \rangle = \frac{1}{\sqrt{2N}} \sum \limits _{j=1}^{N} \left( \mid j; c^j ; d^j \rangle \right) \otimes \left( \mid 0 \rangle + \mid 1 \rangle \right) . \end{aligned}$$
- This step is similar to the one described in Schuld’s proposal to encode the Hamming distance in the amplitude of the corresponding states. Nevertheless, in this case, its purpose is to amplify the probability amplitude of the state with more $\mid 0 \rangle$’s, as those corresponding to the training patterns closest to $x^{k}$.
  Apply the unitary operator $U_f = e^{-i\pi H / 2n}$ to qubits $\mid d^j \rangle$ and the ancilla, where *H* is the operator that sums all the binary digits of $\mid d^j \rangle$. The resulting state is thus
  25 $$\begin{aligned} \mid \psi _3 \rangle = \frac{1}{\sqrt{2N}} \sum \limits _{j=1}^{N} \mid j; c^j \rangle \otimes \left( \sum \limits _{g=0}^{2^n-1} \gamma _g^j \mid g;\phi _0 \rangle \right) , \end{aligned}$$
  where
  26 $$\begin{aligned} \mid \phi _0 \rangle = e^{i\pi z_{g} / 2n} \mid 0 \rangle + e^{-i\pi z_{g} / 2n} \mid 1 \rangle , \end{aligned}$$
  and $z_g$ is the sum of all the binary digits of *g*.
- Lastly, apply a Hadamard gate to the ancilla qubit:
  27 $$\begin{aligned} \mid \psi _4 \rangle = \frac{1}{\sqrt{N}} \sum \limits _{j=1}^{N} \sum \limits _{g=0}^{2^n-1} \gamma _g^j \mid j; c^j; g; \phi _1 \rangle , \end{aligned}$$
  where
  28 $$\begin{aligned} \mid \phi _1 \rangle = \cos \left( \displaystyle \frac{\pi }{2n}z_g \right) \mid 0 \rangle + i \sin \left( \displaystyle \frac{\pi }{2n}z_g \right) \mid 1 \rangle . \end{aligned}$$
  Similar to Schuld’s original algorithm, if the ancilla qubit is in state $\mid 0 \rangle$, there is a high probability of measuring a state corresponding to a pattern close to $x^k$. This occurs because the states corresponding to small values of $z_{g}$, those with more digits equal to 0 in the binary representation of *g*, are the ones corresponding to the nearest neighbors.
  In this case, the probability of finding the ancilla qubit in the state $\mid 0 \rangle$ is
  29 $$\begin{aligned} P_{0} = \frac{1}{N}\sum \limits _{j=1}^{N} \sum \limits _{g=0}^{2^n-1} \left[ \gamma _g^j \cos \left( \frac{\pi }{2n}z_g \right) \right] ^2. \end{aligned}$$
  While the probability of measuring a specific class *C* is given by
  30 $$\begin{aligned} P(C) = \frac{1}{P_0N} \sum \limits _{j\mid x^j\in C}^{N} \sum \limits _{m=0}^{2^n-1} \left[ \gamma _m^j \cos \left( \frac{\pi }{2n}z_m \right) \right] ^{2}. \end{aligned}$$

**Figure 2:**
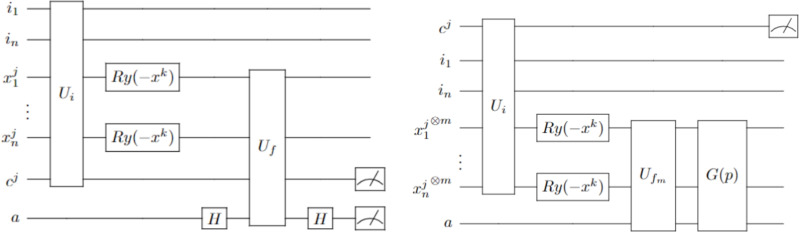
Left: Quantum circuit of Schuld’s QkNN modified algorithm. $U_i$ represents the initialization phase (including training data encoding), $Ry(-x^{k})$ gates represent the comparison rotations, with the data of the unlabeled pattern, and $U_f$ is the gate that implements the evolution of the sum-of-binary-digit Hamiltonian. Right: Quantum circuit of Quezada’s QkNN modified algorithm. $U_i$ represents the initialization phase (including training data encoding), $Ry(-x^{k})$ gates represent the comparison rotations, and $U_{f_m}$ followed by *G*(*p*) form the (*m*, *p*) subroutine.

Analogously, as done in Schuld’s original proposal, the algorithm concludes in one of the two following ways. Firstly, if the ancilla qubit is found to be in $\mid 1 \rangle$, the result is disregarded and counted as one tryout of *t* (the previously defined threshold). Alternatively, if the ancilla measurement yields $\mid 0 \rangle$, the class information qubit is also measured, and the outcome is stored as a class candidate. This process continues until *k* neighbors are obtained or the threshold is reached. At the end, the class that appears the most among the *k* (or less) candidates is selected. Figure 2 shows the circuit associated with Schuld’s modified algorithm.

As previously mentioned, the main advantage of employing the proposed similarity measure lies in reducing required qubits. In contrast to binarized data, which necessitates multiple qubits for each feature, our approach only needs one qubit per feature. To implement this modified version, the number of required qubits (ignoring initialization) is given by

31 $$\begin{aligned} N^{\text {S}}_{\text {modified}} = f+c+1, \end{aligned}$$

where *f* is the number of features in the dataset and *c* represents the number of qubits required to encode the class. Regarding the algorithm’s complexity, the modification only impacts the initialization process, leading to an overall complexity of *O*(*ntN*), which aligns with the original algorithm’s complexity when initialization is considered.

### Quezada’s QkNN modified algorithm

Implementing the similarity measure discussed in Subsect.Metric based on amplitude-encoded features” modifies the QkNN algorithm proposed by Quezada et al. in the following way:

- First, the modified algorithm requires the initial state to be in the following superposition,
  32 $$\begin{aligned} \mid \psi _1 \rangle = \frac{1}{\sqrt{N^{m}}} \sum \limits _{j_{1}, \ldots , j_{m}} \mid c^{j_{1}}; j_{1}, \ldots , j_{m} \rangle \otimes \mid 0 \rangle ^{\otimes n m + 1}, \end{aligned}$$
  where each $j_{i}$ runs from 1 to *N*.
- Apply the controlled rotations $U_{i}$ defined in Eq. (21) to each of the *m* copies of $\mid 0 \rangle ^{\otimes n}$, each one of them controlled by the corresponding register $j_{i}$. Subsequently, implement the similarity between features by applying the rotations $R_{y} \left[ \frac{\pi }{2}\left( -x_{w}^{k}\right) \right]$ in the opposite direction. The resulting state is given by
  33 $$\begin{aligned} \mid \psi _2 \rangle = \frac{1}{\sqrt{N^{m}}} \times \sum \limits _{j_{1}, \ldots , j_{m}} \mid c^{j_{1}}; j_{1}, \ldots , j_{m} ; d^{j_{1}}, \ldots ,d^{j_{m}} \rangle \otimes \mid 0 \rangle , \end{aligned}$$
  where each $\mid d^{j_{i}} \rangle$ is as in Eq. (19).
- Next, apply $U_{f_m}$ the (*m*, *p*) sorting algorithm to sort the states and tag (via the ancilla qubit) those corresponding to the patterns closest to $x^{k}$, following of *G*(*p*) with *p* applications of Grover’s subroutine to amplify the tagged states:
  34 $$\begin{aligned} \mid \psi _3 \rangle = \left[ \frac{\cos [(2p+1)\theta ]}{\sqrt{\nu }} \sum \limits _{J} \sum _{ G \, \text {No ord}} \! \Gamma _{G}^{J} \mid c^{j_{1}}; J; G; \! 0 \rangle \right. + \left. \frac{\sin [(2p+1)\theta ]}{\sqrt{\mu }} \sum \limits _{J} \sum _{ G\,\text {Ord}} \Gamma _{G}^{J}\mid c^{j_{1}}; J; G; \! 1 \rangle \right] . \end{aligned}$$
  Here, in order to simplify the notation, the indexes *J* and *G* respectively denote the sets of indexes $j_{1},\ldots ,j_{m}$ and $g_{1},\ldots ,g_{m}$, such that
  35 $$\begin{aligned} \Gamma _{G}^{J} = \gamma _{g_1}^{j_1} \ldots \gamma _{g_1}^{j_1} \ldots \gamma _{g_m}^{j_m}, \end{aligned}$$
  and
  36 $$\begin{aligned} \mu&= \sum \limits _{J} \sum _{ G \, \text {ord}} \! \mid \Gamma _{G}^{J} \mid ^2 , \end{aligned}$$
  37 $$\begin{aligned} \nu&= \sum \limits _{J} \sum _{ G \, \text {No ord}} \! \mid \Gamma _{G}^{J} \mid ^2 ,\end{aligned}$$
  38 $$\begin{aligned} \theta&= \arcsin \left( \displaystyle \sqrt{\frac{\mu }{N^m}}\right) . \end{aligned}$$
  The probability of measuring a specific class *C* is thus given by
  39 $$\begin{aligned} P(C) = \sum \limits _{J\mid x^{j_{1}}\in C} \left[ \sum _{G \, \text {No ord}} \mid \Gamma _{G}^{J} \mid ^2 + \sum _{G \, \text {ord}} \mid \Gamma _{G}^{J} \mid ^2 \right] . \end{aligned}$$
- As in the original algorithm, we run the previous steps *k* times in order to obtain *k* class candidates. Figure 2 shows the circuit associated with Schuld’s modified algorithm.

Given that the probability distribution in Eq. (19) is structured so that the largest amplitude of the superposition aligns with the Hamming state defined by Eq. (3), we expect the optimal value of *p* to be similar to that of the non-modified version. However, the numerical representation of the features may induce slight variations depending on the dataset.

Like Schuld’s algorithm, the modification here only affects the initialization process, resulting in an overall complexity identical to the original algorithm when initialization is considered. However, the true advantage becomes evident in the memory requirement, as the modified version needs only one qubit per feature. Here, the number of required qubits, without considering initialization, is

40 $$\begin{aligned} N^{\text {Q}}_{\text {modified}} = mf+c+1, \end{aligned}$$

where *f* is the number of features in the dataset and *c* represents the number of qubits required to encode the class.

## Results

**Table 1:** Datasets information: number of patterns, features, classes and imbalance ratio (IR).

Dataset	Patterns	Features	Classes	IR
Iris	150	4	3	1.00
Seed	210	7	3	1.00
Raisin	900	7	2	1.00
Mine	338	3	5	1.09
Cryotherapy	90	6	2	1.14
DBA	1372	4	2	1.25
Caesarian	80	5	2	1.35
Wine	178	13	3	1.48
Haberman	306	3	2	2.78
Transfusion	748	4	2	3.20
Immunotherapy	90	7	2	3.74
Balance scale	624	4	3	5.88
Glass	214	9	7	8.44

**Table 2:** Probabilities of measuring the final qubit as zero and of failing to gather the required *k* candidates in Schuld’s algorithm.

Dataset	$P_0$	$P(\lnot k)$ (percentage %)
Iris	0.65	0.49
Seed	0.60	1.04
Raisin	0.66	0.45
Mine	0.58	1.31
Cryotherapy	0.71	0.19
DBA	0.76	0.08
Caesarian	0.79	0.04
Wine	0.62	0.76
Haberman	0.71	0.19
Transfusion	0.74	0.12
Immunotherapy	0.64	0.60
Balance scale	0.56	1.66
Glass	0.81	0.02

**Table 3:** Accuracy comparison between preprocessing techniches on the classic kNN ($k=1,15$), Schuld’s original and Quezada’s original algorithms, using the Iris dataset and Hamming distance.

Algorithm	Classic 1NN (%)	Classic 15NN (%)	Schuld (%)	Quezada (%)
Scaling, Binary	83.67	88.67	88.00	88.00
Scaling, Gray	91.30	94.67	90.67	90.67
Scaling, Normalized, Binary	87.78	95.33	93.33	94.67
Scaling, Normalized, Gray	91.28	95.33	95.33	95.33

In this section, we conduct a performance comparison of the two QkNN algorithms, including both the original versions and the modified adaptations. The analysis utilizes a set of 13 numerical datasets: Iris ^52^, Cryotherapy ^53^, Seed ^54^, Raisin ^55,56^, Mine ^57,58^, Data Bank Authentication (DBA) ^59^ and Caesarian ^60^, which are balanced datasets, as well as Wine ^61^, Haberman ^62^, Transfusion ^63^, Immunotherapy ^64,65^, Balance scale ^66^, and Glass ^67^, which are imbalanced datasets. Detailed information regarding these datasets, including their imbalance ratio (IR), can be found in Table 1.

### Methodology

All the data analysis here presented was performed using Python 3.12: the scikit-learn library to evaluate performance metrics and Qiskit to simulate algorithms and assess noise. The computations were carried out on a system with the following specifications: an Intel Core i7 10700K CPU at 3.80GHz, 48GB of RAM, and an Nvidia GeForce RTX 2060S GPU.

For the performance comparison, we employ two metrics: Accuracy and F1 score. Accuracy represents the proportion of correctly classified patterns out of the total predictions, and it is widely used for evaluating classification models. However, for imbalanced datasets, accuracy may not provide reliable results. In such cases, utilizing the F1 score, defined as the harmonic mean of precision and recall, is recommended. In addition, following the common practice in the performance analysis of quantum machine learning algorithms, we employ the Leave-One-Out method as the validation method. This deterministic approach ensures that no additional probabilistic factors impact the outcome, providing reliable and consistent results.

Data preprocessing is a crucial step in classification algorithms, as it can significantly enhance their performance. Here, various tests were conducted to analyze the performance of both QkNN algorithms, including normalization of numerical values and different types of binary encoding (Table 3). In the case of the original versions, we found that the best results were obtained using normalization and scaling of the datasets to integers, followed by binarization using the Gray code, which is explicitly designed for numerical data. On the other hand, for the modified algorithms, preprocessing solely consisted on normalizing the numerical data to have values between 0 and 1. This preprocessing techniques were applied to all datasets to ensure a fair comparison of results, guaranteeing that the only factor influencing performance was the algorithms and their corresponding metric.

For Schuld’s algorithm, we set the threshold value at $T=5k$. The resulting probability of measuring the last qubit as $\mid 0 \rangle$ and the probability of not gathering the required *k* candidates at the algorithm’s conclusion ($P(\lnot k)$) are presented in Table 2 for each dataset. As for Quezada’s original algorithm, when $m=2$, the optimal value of *p* is found to be $p_{\text {opt}} = 0.5$ regardless of the dataset (as long as *N* is large enough). However, as we have stated before, in the modified version, the optimal parameter is expected to exhibit slight variations depending on the dataset. Consequently, we conduct our analyses using $m=2$ in all cases and a range of *p* values, specifically $p \in \{0.5, 1, \dots , 8\}$. It is important to note that the case $p=0.5$ introduces a minor complication, as *p* is defined as a natural number. Nevertheless, from a strict mathematical perspective, $p=0.5$ also defines a valid unitary operator, and thus we employ it for testing purposes.

The maximum accuracy and F1-score obtained for each dataset and version of QkNN are presented in Tables 4 and 5, respectively. In most cases, this value was obtained utilizing a theoretical approach, assigning to each pattern the class with the maximum probability, calculated using Eqs. (5),  (12),  (30) and  (39). However, for some datasets, the maximum performance was obtained through experimentation and for a finite value of k.

**Figure 3:**
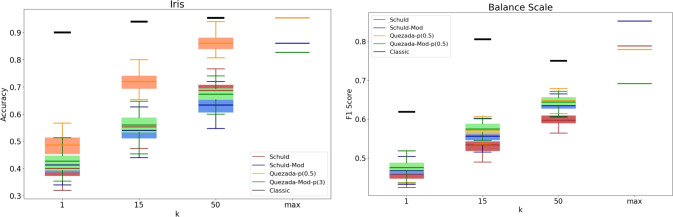
Accuracy results for the Iris (left) and F1 score for balance scale (right) datasets using the algorithms: Schuld, Schuld-Mod, Quezada and Quezada-Mod with $\hbox {m} = 2$.

**Figure 4:**
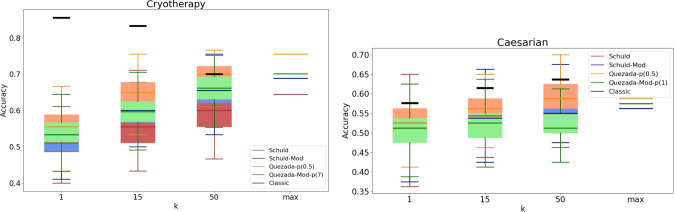
Accuracy results for the Cryotherapy (left) and Caesarian (right) dataset using the algorithms: Schuld, Schuld-Mod, Quezada and Quezada-Mod with $\hbox {m} = 2$.

**Figure 5:**
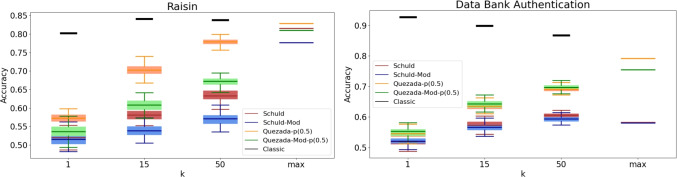
Accuracy results for the Raisin (left) and DBA (right) dataset using the algorithms: Schuld, Schuld-Mod, Quezada and Quezada-Mod with $\hbox {m} = 2$.

**Figure 6:**
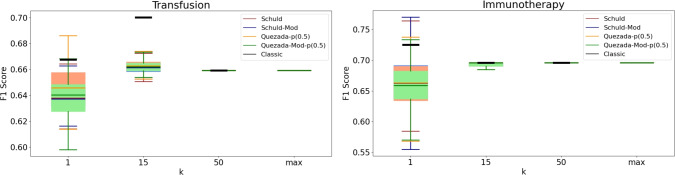
F1 score results for the transfusion (left) and immunotherapy (right) dataset using the algorithms: Schuld, Schuld-Mod, Quezada and Quezada-Mod with $\hbox {m} = 2$.

**Figure 7:**
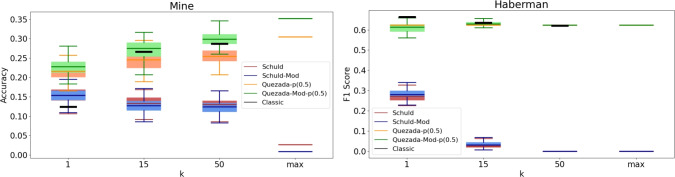
Accuracy results for the mine (left) and F1 score for Haberman (right) dataset using the algorithms: Schuld, Schuld-Mod, Quezada and Quezada-Mod with $\hbox {m} = 2$.

**Figure 8:**
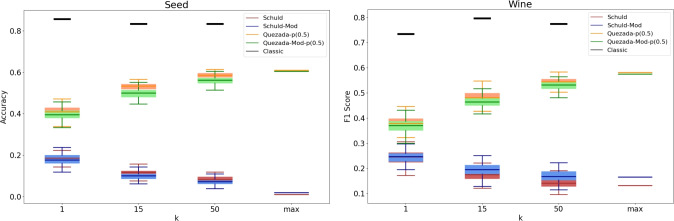
Accuracy results for the seed (left) and F1 Score for the wine (right) datasets using the algorithms: Schuld, Schuld-Mod, Quezada and Quezada-Mod with $\hbox {m} = 2$.

**Figure 9:**
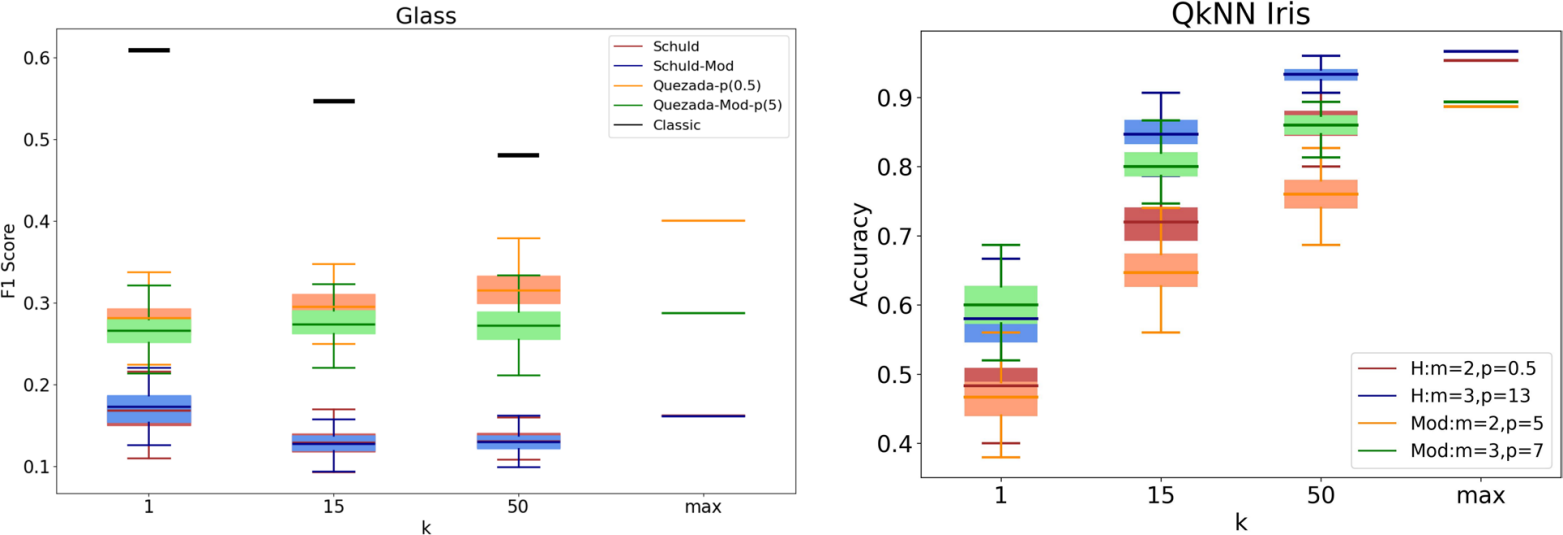
F1 score results for the glass dataset using the algorithms: Schuld, Schuld-Mod, Quezada and Quezada-Mod with m = 2. Accuracy results for the Iris (right) dataset using Quezada’s original and modified algorithms with $\hbox {m} = 2$ and $\hbox {m} = 3$.

It is important to recognize that a practical implementation of these algorithms may produce different results. To analyze the practical behavior of the algorithms, we conducted a series of experiments with specific values for *k*, specifically $k \in \{1, 15, 50\}$. Given the inherently probabilistic nature of quantum algorithms, we repeated these experiments 100 times for each value of *k*, resulting in an accuracy (or F1-score) distribution in each case. The findings are presented in Figs. 3, 4, 5, 6, 7, 8, and 9, illustrated as box-whisker plots for all datasets.

**Table 4:** Accuracy.

Dataset	Schuld	Schuld-Mod	Quezada	Quezada-Mod
Iris	0.9533	0.8600	0.9533	0.8267 ($p=3$)
Seed	**0.1866**	**0.1764**	0.6095	0.6095 ($p=0.5$)
Raisin	0.8156	0.7767	0.8289	0.8100 ($p=0.5$)
Mine	**0.1557**	**0.1539**	0.3047	0.3521 ($p=0.5$)
Cryotherapy	0.6444	0.6889	0.7556	0.7012 ($p=7$)
DBA	**0.6096**	**0.5937**	0.7915	0.7551 ($p=0.5$)
Caesarian	0.5750	0.5625	0.5956	0.5750 ($p=1$)
Wine	**0.2267**	**0.2391**	0.6742	0.6798 ($p=0.5$)
Haberman	**0.1830**	**0.1943**	0.7353	0.7451 ($p=0.5$)
Transfusion	0.7621	0.7620	0.7620	0.7673 ($p=0.5$)
Immunotherapy	0.7889	0.7889	0.7889	0.7889 ($p=0.5$)
Balance scale	0.8224	0.8896	0.8112	0.7216 ($p=0.5$)
Glass	**0.3271**	**0.3271**	0.4907	0.3925 ($p=5$)

Values in bold represent the average for cases where the maximum performance was achieved experimentally for a finite value of k.

**Table 5:** F1 score.

Dataset	Schuld	Schuld-Mod	Quezada	Quezada-Mod
Iris	0.9533	0.8593	0.9533	0.8267 ($p=3$)
Seed	**0.2333**	**0.1794**	0.5103	0.5100 ($p=0.5$)
Raisin	0.8125	0.7668	0.8278	0.8051 ($p=0.5$)
Mine	**0.1585**	**0.1554**	0.2576	0.3284 ($p=0.5$)
Cryotherapy	0.5795	0.6523	0.7384	0.6724 ($p=7$)
DBA	**0.5624**	**0.5520**	0.7813	0.7312 ($p=0.5$)
Caesarian	0.5271	0.5374	0.5822	0.6889 ($p=1$)
Wine	**0.2483**	**0.2494**	0.5808	0.5759 ($p=0.5$)
Haberman	**0.2724**	**0.2864**	0.6231	0.6239 ($p=0.5$)
Transfusion	0.6621	0.6619	0.6634	0.6776 ($p=0.5$)
Immunotherapy	0.6958	0.6958	0.6958	0.6958 ($p=0.5$)
Balance Scale	0.7889	0.8533	0.7797	0.6921 ($p=0.5$)
Glass	**0.1612**	**0.1618**	0.4004	0.2879 ($p=5$)

Values in bold represent the average for cases where the maximum performance was achieved experimentally for a finite value of k.

### Discussion

From Eqs. (6), (17), (31), and (40), it is evident that the reduction in the number of required qubits when using the modified algorithms can be significant in most real-life scenarios. Here, we analyze whether this benefit comes at the cost of performance.

The theoretical results in Tables 4 and 5 indicate that Schuld’s algorithm shows significant improvements in Cryotherapy and Balance Scale, but exhibits reduced performance in Iris and Raisin, while maintaining similar results in the remaining nine datasets. Conversely, Quezada’s algorithm demonstrates better performance in Caesarian, Haberman, and Immunotherapy, but performs worse in Iris, Balance Scale, and Glass, while maintaining similar results in the remaining seven datasets.

Regarding real-life performance, experiments on the Iris dataset (left panel of Fig. 3) show that both modified versions exhibit performance comparable to Schuld’s original algorithm for the analyzed values of *k*. In contrast, Quezada’s original algorithm consistently achieves the highest accuracy in all cases. However, it is important to note that the theoretical maximum accuracy of both original versions exceeds that of the modified versions.

The case of the Balance Scale dataset (right panel of Fig. 3) is particularly interesting. Here, for the studied values of *k*, the performance of the four algorithms is quite similar, with one exception: Schuld’s original version lags slightly behind when $k=50$. Nevertheless, when we consider the theoretical maximums, Schuld’s modified version achieves the highest F1-score, followed by the original version. In contrast, the modification has an adverse effect on Quezada’s algorithm, resulting in Quezada’s modified version delivering the lowest performance among the four.

In the Cryotherapy dataset (left panel of Fig. 4), the observed behavior for finite values of *k* closely aligns with the behavior at the theoretical limit. In both cases, the performance of the modified algorithms falls between that of their original counterparts. Here, Schuld’s original algorithm consistently performs the worst, while Quezada’s original algorithm delivers the best results. Notably, the mean accuracy values for both modified versions are similar; however, it is worth noting that the accuracy distribution for Quezada’s modified version exhibits a narrower spread across all finite cases.

The experiments on the Caesarian dataset (right panel of Fig. 4) exhibit a consistent performance trend across all four quantum algorithms, with performance steadily and slowly increasing as *k* increases. However, the results also show a highly dispersed distribution, suggesting significant overlap among classes in the feature space. Interestingly, although Caesarian’s imbalance ratio (IR) is not high enough to be considered an imbalanced dataset, the theoretical F1 score gives a significant advantage to Quezada’s modified version. Unfortunately, this advantage does not manifest for the analyzed values of *k*.

In the Raisin dataset (left panel of Fig. 5), all algorithms show a steadily increasing performance as *k* increases. However, they do so at different rates, which is crucial for real-life implementations, as larger *k* values result in longer processing times for quantum algorithms. In this case, Quezada’s original algorithm leads, reaching near-maximum performance at $k=50$. In the DBA dataset (left panel of Fig. 5), a similar pattern is observed. However, in this case, both Quezada’s original and modified algorithms show a comparable rate of performance improvement, with the modified version demonstrating slight superiority for the analyzed values of *k*.

Experiments on both the Transfusion and Immunotherapy datasets reveal interestingly similar results (Fig. 6) for all algorithms, with all reaching their maximum theoretical performance at relatively low values of *k*. Furthermore, the performance distribution of all four algorithms converged to a single value, showing no advantage for any specific metric or approach. This behavior can be attributed to one class having a significantly higher probability of being assigned to every pattern (usually the majority class), which also explains why the classical algorithm achieves the same performance as its quantum counterparts for large values of *k* ($k=50$ in this case).

Results on the Mine dataset (left panel of Fig. 7) show poor performance with all algorithms, consistent with the classical results. This suggests that the dataset is not well-suited for analysis using distance-based algorithms. Quezada’s more complex approach, compared to Schuld’s, is evident as performance increases with *k* for both the modified and original versions, even in this challenging dataset.

Similar to the Transfusion and Immunotherapy datasets, the Haberman dataset (right panel of Fig. 7) also exhibits a performance distribution with low spread, even for $k=1$, eventually converging to a single value at $k \le 50$. However, in this case, Quezada’s approach demonstrates its capabilities, as both the original and modified versions outperform Schuld’s algorithms, suggesting the metric itself was not a relevant feature in the analysis of this dataset.

In both the Seed and Wine datasets (Fig. 8), the performance of both versions of Schuld’s algorithm is strikingly deficient, even decreasing as *k* increases. In contrast, Quezada’s algorithm behaves as expected, whether in its modified or original form, with the mean accuracy values increasing as *k* grows. Both versions display similar behavior across all scenarios, with the original algorithm producing slightly higher mean accuracy values.

Lastly, in the case of the Glass dataset, for finite values of *k*, the performance of both versions of Schuld’s algorithm and the modified version of Quezada’s algorithm appears to stall without substantial improvement as *k* increases. This pattern remains consistent even when considering the theoretical maximum values, which closely resemble the mean F1-score obtained for $k=1$. In this dataset, only Quezada’s original version behaves as expected by consistently enhancing its performance as *k* increases, ultimately achieving the highest overall performance among the algorithms.

One of the key differences between the classical and quantum versions of the KNN algorithm is the interpretation of the parameter *k*. While in the classical algorithm, *k* represents the number of (strictly different) neighbors considered for class selection, the quantum algorithms behave as a probabilistic version of the classical 1NN algorithm, randomly (weighted by the corresponding probability distribution) picking one neighbor in each execution. Hence, the performance of the quantum versions will increase with *k* if the maximum obtained probability corresponds to the correct class. Conversely, if this is not the case, performance will decrease with *k*. This feature explains the behavior observed in the analysis of some datasets, as Quezada’s algorithm, which sacrifices simplicity for performance, is more likely to obtain a maximum probability for the correct class. On the other hand, Schuld’s algorithm sacrifices performance for simplicity, making it more prone to obtain a maximum probability for the wrong class.

Throughout this analysis, for both versions of Quezada’s algorithm, we have used $m=2$. The reason for this choice is that the computation time required for simulating the quantum algorithm on a classical computer significantly increases with higher values of *m*, particularly in the case of the modified version, as can be inferred from Eq. (39). In the right panel of Fig. 9, we compare the performance achieved with $m=2$ and $m=3$ on the Iris dataset. Here, we observe that increasing the value of *m*, and consequently the value of *p*, results in a steeper rise in performance as *k* increases. For each finite value of *k* the accuracy of both algorithms with $m=3$ outperforms their counterparts with $m=2$. Interestingly, this pattern holds even for the theoretical maximum, even though the performance improvement is less than 1.5%. Regarding the proposed metric, we observe that the modified versions perform similarly to their original counterparts for $k=1$. However, as *k* increases, the performance of the original versions surpasses that of the modified versions, consistent with the pattern observed in the left panel of Fig. 3 for the Iris dataset.

### Noise analysis

In order to assess the possible effect that noise would have on the performance of the modified algorithms compared to the original versions, we simulated a noisy implementation using the Qiskit AerSimulator simulator, which introduces depolarization errors for one-, two-, and three-qubit gates.

**Table 6:** Accuracy obtained from 2048 executions for each algorithm with and without noise.

Algorithm	Without noise (%)	Noise (%)
Schuld	78.46	70.28
Schuld Mod	83.92	66.98
Quezada	69.23	53.97
Quezada Mod	62.53	53.14

For this purpose, a prototype dataset of four patterns, two features and two classes, was created. Using this toy-model dataset, the implementation of Schuld’s original algorithm required 10 qubits, while the modified version required only 6. Similarly, Quezada’s original algorithm (with $m=2$) utilized 14 qubits, whereas the modified version needed only 10. Thus demonstrating the advantage of the modified versions in reducing the number of required qubits.

Table 6 show the results obtained from 2048 executions. The noisy implementation of Shuld’s and Quezada’s original algorithms decreased the accuracy by $8.18\%$ and $15.26\%$ respectively. On the other hand, the corresponding reduction in both Shuld’s and Quezada’s modified algorithms was $16.94\%$ and $9.39\%$ respectively.

These mixed results clearly indicate that, for Schuld’s algorithm, the modified version is more susceptible to depolarization than its original counterpart. In contrast, for Quezada’s algorithm, the modified version proved to be more resilient. In both cases, susceptibility to depolarization was observed during the initialization phase, where the noise induced by the QRAM impacted the performance of both modified algorithms. However, the results suggest that Quezada’s original initialization phase is more prone to noise-induced errors than Schuld’s.

## Conclusions

In this work, we introduced a quantum similarity measure for patterns and integrated it into two quantum adaptations of the kNN algorithm. To evaluate the impact of this modification, we conducted benchmark tests on both the original and modified versions of these algorithms across 13 diverse datasets (Iris, Seeds, Raisin, Mine, Cryotherapy, Data Bank Authentication, Caesarian, Wine, Haberman, Transfusion, Immunotherapy, Balance Scale, and Glass). The main advantages of this implementation encompass the use of non-binarized numerical data and a reduced memory requirement when compared to the original versions, all while maintaining their complexity.

Both theoretical and real-life results show that both algorithms benefit from the proposed subroutine, achieving a considerable reduction in the number of required qubits, while maintaining a similar overall performance. It is expected that not all datasets will show improvement, as the “no-free-lunch” theorem ^68^ states that no classifier delivers good results for all datasets. The modified versions can thus be considered the first choice for analyzing datasets similar to those where they showed improved performance, and be regarded as a memory-efficient option for analyzing datasets where they did not show improvement.

This study highlights the dynamic nature of quantum machine learning and the need for adaptable quantum algorithms. The contrasting outcomes for Schuld’s and Quezada’s algorithms illustrate the intricate interplay between quantum techniques and the specific characteristics of datasets. These results reinforce the notion that quantum machine learning is an evolving discipline where choices must be made according to the distinctive requirements of each application. Future studies should focus on developing and optimizing quantum algorithms for various datasets, ensuring that quantum machine learning continues evolving as a powerful data analysis tool.

## Data Availability

The datasets analysed during the current study are available in the UC Irvine machine learning repository: Iris - https://doi.org/10.24432/C56C76. Seeds - https://doi.org/10.24432/C5H30K. Raisin - https://doi.org/10.24432/C5660T. Land Mines - https://doi.org/10.24432/C54C8Z. Cryotherapy - https://doi.org/10.24432/C5FC7C. Banknote Authentication - https://doi.org/10.24432/C55P57. Caesarian Section Classification - https://doi.org/10.24432/C5N59X. Wine - https://doi.org/10.24432/C5PC7J. Haberman’s Survival - https://doi.org/10.24432/C5XK51. Blood Transfusion Service Center - https://doi.org/10.24432/C5GS39. Immunotherapy - https://doi.org/10.24432/C5DC72. Balance Scale - https://doi.org/10.24432/C5488X. Glass Identification - https://doi.org/10.24432/C5WW2P.
